# Comparisons of Respiratory Function and Cardiorespiratory Responses Induced by the Modified Shuttle Walk Test in Children Finswimmers and Age-Matched Sedentary Non-Athletes

**DOI:** 10.3390/jcm15072806

**Published:** 2026-04-07

**Authors:** Theano Michailidou, Aspasia Mavronasou, Eleni A. Kortianou

**Affiliations:** Clinical Exercise Physiology and Rehabilitation Laboratory, Physiotherapy Department, University of Thessaly, 35100 Lamia, Greece; tmichailidou@uth.gr (T.M.); asmavronasou@uth.gr (A.M.)

**Keywords:** cardiorespiratory fitness, children, finswimmers, modified shuttle walk test, non-athletes, respiratory function

## Abstract

**Background**: Regular aerobic exercise during childhood promotes critical physiological adaptations in the cardiovascular and respiratory system. Finswimming, a unique aquatic sport, requires high-intensity demands and specific breathing patterns. The present study aimed to compare respiratory function and cardiorespiratory responses between young male finswimmers and sedentary age-matched non-athletes. **Methods**: Thirty-two boys aged 8 to 12 years old were stratified into the finswimmers group (FSG, n = 16) and the non-athletes group (NAG, n = 16). Assessments included pulmonary function (spirometry) and respiratory muscle strength (Maximum Inspiratory Pressure, MIP/Maximum Expiratory Pressure, MEP). Exercise capacity was evaluated using the modified shuttle walk test (MSWT). **Results**: The FSG exhibited significantly higher pulmonary function (Forced Vital Capacity, Forced Expiratory Volume in 1 s, Maximum Voluntary Ventilation; *p* < 0.05) and superior MIP compared to the NAG (105.3 ± 24.8 versus 87.3 ± 24.7 cmH_2_O; *p* = 0.022). During the MSWT, FSG covered substantially greater distances (746.6 ± 97.2 versus 591.1 ± 86.4 m; *p* < 0.001) with lower levels of leg fatigue (Borg 0–10) (0.53 ± 0.39 versus 2.13 ± 1.93; *p* = 0.004) and demonstrated lower heart rate recovery time (4.47 ± 0.68 versus 5.75 ± 0.68 min; *p* < 0.001) compared to NAG. At the iso-level (8th level of MSWT), FSG scored lower levels of leg fatigue (0.13 ± 0.12 versus 2.02 ± 2.0; *p* = 0.001) compared to NAG, indicating better peripheral oxygen % saturation (100 ± 0.0 versus 98.14 ± 1.16; *p* < 0.001). **Conclusions**: Systematic exercise training enhances profound cardiorespiratory and peripheral muscle adaptations in children. Enhanced cardiorespiratory function allows young athletes to achieve higher workloads and recover faster than sedentary peers, highlighting the sport’s role in establishing a robust cardiorespiratory fitness.

## 1. Introduction

Regular aerobic exercise induces profound physiological adaptations that enhance cardiovascular and respiratory function, including increased cardiac output, improved oxygen uptake, greater efficiency of pulmonary ventilation (enhancing gas exchange), and strengthens respiratory muscles [1]. Cardiovascular adaptations include increased myocardial contractility, improved stroke volume, and enhanced vascular compliance [2]. Furthermore, systematic exercise training in childhood promotes favorable cardiac remodeling and improves autonomic nervous system regulation, often evidenced by increased heart rate variability and more efficient heart rate recovery dynamics [3]. Prospective studies have demonstrated that high levels of cardiorespiratory fitness (CRF) in youth are strongly associated with a more favorable cardiovascular profile in adulthood [4]. In addition, higher levels of physical activity led to superior physical fitness and enhanced performance in daily activities, serving as a critical indicator of health status for the pediatric population [5]. Conversely, diminished cardiorespiratory capacity in children negatively impacts endurance, muscular strength, and overall quality of life, thereby escalating the risk of cardiovascular events, such as myocardial infarction, and contributing to premature mortality in adult life [6]. Despite the aforementioned benefits, approximately 80% of children worldwide are currently physically inactive, leading to a “fitness gap” between active and sedentary populations [7].

Recent research indicates that children aged 8 to 11 who participate in exercise programs, regardless of specialization in a specific sport, exhibit superior CRF compared to their sedentary peers [8]. In particular, young athletes (aged 7 to 12 years) involved in swimming and tennis demonstrate greater aerobic capacity than non-athletes, mainly due to differences in muscle mass observed in athletes [9]. These differences are primarily attributed to the physiological adaptations resulting from systematic physical training. Young swimmers often possess larger chest circumference, greater lean body mass, and increased muscle cross-sectional area in the upper arms as a result of systematic training, factors which ultimately enhance their performance in functional evaluation tests [10]. Among sports, swimming and finswimming are unique, as they demand high-intensity aerobic engagement while simultaneously challenging the respiratory system through controlled breathing patterns [11]. According to Wylegala et al. [11], this dual demand creates a significant load on the respiratory muscles due to the added hydrostatic pressure of the equipment used (snorkels). Their research demonstrates that when these muscles are specifically trained to handle such challenges, swimming endurance can improve by up to 33%, highlighting that respiratory efficiency is just as critical as cardiovascular power in aquatic environments. Previous studies have shown that children participating in such organized sports exhibit not only superior lung volumes (Forced Vital Capacity, FVC) but also enhanced cardiovascular efficiency (higher oxygen uptake, lower heart rate, and peak blood lactate concentrations) compared to their sedentary peers [12].

Although assessment of CRF through maximal oxygen uptake is considered the gold standard in pediatric exercise physiology [13], laboratory-based cardiopulmonary exercise testing is often impractical due to high costs and specialized equipment requirements [14]. Consequently, field-based tests have been developed as valid surrogates for clinical and school settings. The 20-m Shuttle Run Test (20m-SRT) has been extensively used as a health surveillance tool [15], yet it frequently presents pacing difficulties for younger children, where the inability to synchronize speed with acoustic signals may result in performance that does not reflect true physiological limits [16]. To mitigate these cognitive and motivational barriers, incremental shuttle walk tests, such as the modified Shuttle Walk Test (MSWT), offer a more structured and progressive approach to workload elevation [17]. The MSWT is particularly advantageous for comprehensive cardiovascular profiling in both healthy and disease pediatric populations [18,19]. Its incremental nature allows for the monitoring of cardiorespiratory responses in a way that is both reliable and reproducible within the pediatric population [20] and can be used as an alternative to evaluating exercise capacity [19,21]. By eliminating complex pacing strategies, the MSWT ensures that the observed functional capacity is a more accurate reflection of the child’s actual cardiovascular reserve [19]. To the best of our knowledge, MSWT has not previously been used to assess CRF in children finswimmers.

Given the importance of evaluating cardiorespiratory responses and respiratory function in this population, this study aimed to compare pulmonary function, respiratory muscle strength, and cardiorespiratory indices during an incremental walk test in a pediatric cohort of boy finswimmers and compare with those of age-matched non-athletes.

## 2. Materials and Methods

### 2.1. Participants

This comparative study, conducted from December 2024 to March 2025, aimed to evaluate pulmonary function, respiratory muscle strength, modified Shuttle Walk Test (MSWT) performance, and associated cardiorespiratory responses in healthy sedentary children versus finswimmers. A purposive sample of children aged 8 to 12 years was recruited from the Dolfins Swim Academy and various community clubs in Xanthi, Greece. The cohort was stratified into two age-matched groups: the finswimmers group (FSG) and the non-athletes group (NAG). Participants met the following inclusion criteria: (a) healthy boys, (b) aged between 8 and 12 years old, (c) finswimmers, and (d) non-athletes. The exclusion criteria were: (a) participation in sports, (b) presence of cardiorespiratory, musculoskeletal, and neurological diseases, (c) children with developmental disorders, (d) inability to perceive and follow instructions of the assessment process, and (e) inability to understand and use the Greek language. The parents/caregivers of the children were fully informed of the aims of the study and provided written informed consent. The study protocol followed the ethical standards of the World Medical Association (Declaration of Helsinki), and it was approved by the Ethics Committee of the Physiotherapy Department, University of Thessaly (Protocol ID 18920/24).

### 2.2. Study Design

This was a cross-sectional, comparative, non-blinded study. Our primary aims were to assess pulmonary function, respiratory muscle strength, and exercise capacity of both FSG and NAG. All measurements were performed on two consecutive days by the same researcher (T.M.), in a standardized testing indoor environment. A second independent examiner (A.M.) was present to ensure data quality.

On the first day, anthropometric and body composition measurements were performed. Height was measured using a fixed wall stadiometer (seca GmbH & Co. KG, Hamburg, Germany), with children barefoot and standing upright, following standard procedures [22]. Body composition (weight, body mass index, body fat, and muscle mass) was evaluated using the TANITA RD-545 bioelectrical analyzer (TANITA Corporation, Tokyo, Japan), according to the manufacturer’s protocol [23]. On the same day, pulmonary function was assessed using a portable spirometer (MIR-Spirobank II Basic, MIR Medical International Research, Rome, Italy). Standardized instructions were provided according to international guidelines, and a nose clip was used to avoid air leakage [24]. Respiratory muscle strength (Maximum Inspiratory Pressure, MIP/Maximum Expiratory Pressure, MEP) was measured using the MicroRPM digital mouth pressure device (Micro Medical Ltd, Chatham, United Kingdom) [25]. Handgrip strength was assessed using a Kinvent K-GRIP dynamometer (Kinvent Biomecanique, Montpellier, France) in a seated position, with no armrests and stable lower limb support [26].

On the second day, children performed the MSWT. Before testing, children underwent a 30-min rest period to mitigate any exertional fatigue associated with transit. All children were instructed to wear appropriate athletic clothing and footwear. Two trials were performed, separated by 30 min of rest, and the best performance (greater distance covered) was considered for analysis.

### 2.3. Measurements

#### 2.3.1. Pulmonary Function

Pulmonary function was evaluated using the MIR-Spiro Lab portable Spirometer (MIR—Medical International Research, Rome, Italy) and following the instructions of the European Respiratory Society guidelines [24]. Absolute and % of predicted values for the forced expiratory volume in one second (FEV_1_), forced vital capacity (FVC), peak expiratory flow (PEF), the FEV_1_/FVC ratio, and maximal voluntary ventilation (MVV) were assessed in the sitting position for each child.

#### 2.3.2. Respiratory Muscle Strength

Maximal inspiratory pressure (MIP) and maximal expiratory pressure (MEP) were measured using the MicroRPM digital mouth pressure device (CareFusion, San Diego, CA, USA). Assessments were conducted in accordance with the American Thoracic Society (ATS) guidelines [24]. During the procedure, children were seated in an upright position with the trunk, hips, and knees maintained at approximately 90° of flexion.

To measure MEP, they were instructed to inspire to total lung capacity (TLC) and subsequently execute a maximal forced expiration through the mouthpiece for a minimum duration of 2 s. Conversely, MIP was assessed by requiring children to exhale to residual volume (RV), followed by a maximal, forceful inspiration sustained for at least 2 s. To prevent air leakage, a disinfected nasal clip was applied, and children were instructed to maintain an airtight seal around the mouthpiece throughout each maneuver.

A minimum of three maneuvers were recorded for both MIP and MEP, with the highest achieved values selected for subsequent analysis. In addition to absolute values, the percentage of predicted value was calculated using the reference equation by Heinzmann-Filho et al. [27]. To mitigate the confounding effects of respiratory muscle fatigue, a 1-min recovery period was provided between individual trials, while a 5-min rest interval was maintained to separate the inspiratory and expiratory measurement sets [28].

#### 2.3.3. Exercise Capacity

The MSWT was administered according to the standardized protocol originally described by Singh et al. [17] and later adapted for healthy populations to minimize the ceiling effect [29]. The test was conducted on a 10-m course marked by two cones placed 0.5 m from each endpoint. The test consisted of 15 progressive levels, with the walking speed beginning at 0.5 m/s and increasing by 0.17 m/s per minute. Level transitions and speed increments were indicated by standardized audio “beeps” [30]. Children were instructed to walk in time with the auditory signals from the metronome, ensuring they reached each cone precisely as the beep sounded. Children were monitored by the physiotherapist during the test for adaptation to the rhythm of the audio signal. The physiotherapist did not provide any verbal encouragement during testing [17,31].

To monitor cardiorespiratory parameters, heart rate (HR), blood pressure (BP), pulse oxygen saturation (SpO_2_), dyspnea, and leg fatigue (BORG scale 0–10) [32] were measured at the beginning, at the end, and for the next 6 min after the test (recovery period in sitting position). BP was measured using an Omron M2 sphygmomanometer (OMRON Healthcare Co., Ltd, Kyoto, Japan) with a pediatric cuff. HR, SpO_2_, dyspnea, and leg fatigue were recorded at every speed level during walking, and at each minute of the recovery period. Before starting testing, children were seated to achieve resting conditions, and all cardiorespiratory parameters were recorded. The test was terminated under one of the following conditions: (a) completion of all levels, (b) failure to maintain the required pace (i.e., being more than 0.5 m from the cone for two consecutive beeps), (c) achievement of predicted maximal heart rate (HRmax), or (d) onset of dyspnea or fatigue [33,34].

Each child performed the MSWT twice, separated by a 30-min rest interval, following the standardized guidelines proposed by Singh et al. [34]. The best performance (longest distance covered) was included in the analysis. The predicted distance (in meters) derived from the reference equation proposed by Lanza et al. [30].

#### 2.3.4. Body Composition

Body composition was assessed using a TANITA RD-545 bioelectrical impedance analyzer (Tanita Corp., Tokyo, Japan). The device was positioned on a firm surface, and children stood barefoot on the electrodes while holding the hand grips with extended arms, remaining still during the measurement [23]. Body mass index (BMI) in kg/m^2^, fat-free mass (FFM) in kg, fat-free mass index (FFMI) in kg/m^2^, and body fat percentage (%BF) were extracted.

#### 2.3.5. Handgrip Strength

Handgrip strength (in kg) was measured using Kinvent K-GRIP (Kinvent, Montpellier, France) in a standardized seated position without arm support. Children were sitting with hips and knees flexed at approximately 90°, shoulders adducted and neutrally rotated, and elbows flexed at 90°. The forearm was in a neutral position, and the wrist maintained 15–30° of extension with 0–15° of ulnar deviation. Three trials for each upper limb were performed, and the highest effort in Kg was used for the analysis. External stabilization was provided by the examiner to minimize compensatory movements [26].

#### 2.3.6. Physical Activity Level

The Physical Activity Questionnaire for Older Children (PAQ-C) was used to assess physical activity levels [35]. The PAQ-C is a 10-item, self-reported questionnaire, designed for students aged 8 to 14, that records an individual’s physical activity (PA) over the past 7 days. Nine of the ten items are scored on a five-point Likert scale, with higher scores reflecting increased activity levels. The first item of the PAQ-C consists of 22 common sports and leisure activities for which children rate the frequency of the activities performed during the previous week (1 = no activity at all, 2 = 1–2 times, 3 = 3–4 times, 4 = 5–6 times, and 5 = 7 times or more) after a mean composite score is calculated. The remaining eight items measure PA across specific daily settings (e.g., physical education classes, recess time, lunch, as well as after-school hours, weekends, and a summary for all days of the week. The final PAQ-C summary score is determined by calculating the average of the nine individual item scores. The highest and lowest values are 1 and 5, respectively.

### 2.4. Statistical Analysis

A power analysis was performed based on previous data [18], which reported a standard deviation of 202.8 m for the MSWT in healthy pediatric populations. To achieve 80% statistical power with a significance level of α = 0.05, a minimum sample of 24 children was required. Assuming a dropout rate of 20%, the target sample size was estimated at thirty children.

Statistical analysis was performed using the IBM SPSS for Mac, version 29.0 (SPSS Inc., Chicago, IL, USA). The Shapiro–Wilk test was used to test the normality of the distribution of the data. Categorical variables were reported as numbers (and percentages). Continuous variables were reported as means ± standard deviation (SD), and medians (interquartile ranges, IQR), depending on the normality of distribution. The independent *t*-tests and Mann–Whitney U-test were used for comparisons between groups. The correlations between variables were analyzed using Pearson (r) and Spearman (ρ) correlation coefficients. Interpretations of correlations were based on Cohen’s guidelines: values from 0.10 to 0.29 indicate a small correlation, 0.30 to 0.49 as medium, and 0.50 to 1.00 as a large correlation [36]. Statistical significance was defined as *p* < 0.05.

## 3. Results

Thirty-two children who met the inclusion criteria and were willing to participate were included in the study. The final sample consisted of 32 eligible participants, comprising two equal cohorts of athletes (n = 16) and non-athletes (n = 16). No statistically significant differences were observed between FSG and NAG in age, height, weight, BMI, or body fat percentage. The athletes participating in finswimming reported an average training experience of approximately 2.5 ± 1 years (Table 1).

Data derived from the PAQ-C revealed a significant discrepancy in lifestyle habits between the two cohorts. The FSG demonstrated substantially higher PA scores compared to the NAG (*p* = 0.003), indicating a consistently higher volume of moderate-to-vigorous physical exertion (Table 1).

The majority of participants were right-handed (n = 30), with only two participants identifying as left-handed, one in each group. Assessment of handgrip strength in kilograms showed that the FSG recorded higher mean values for both the right and the left hand with no statistically significant differences between groups.

The anthropometric characteristics, physical activity, and handgrip strength of the two groups are summarized in Table 1.

The respiratory function of the FSG and the NAG groups is summarized in Table 2. The FSG exhibited significantly enhanced pulmonary function compared to the NAG, characterized by marked increases in both absolute and relative values in all pulmonary parameters, except peak expiratory flow (PEF) % predicted. Furthermore, MIP was significantly higher in the FSG cohort. In contrast, no statistically significant differences were observed between groups regarding MEP absolute or % predicted values (*p* > 0.05).

**Figure 1:**
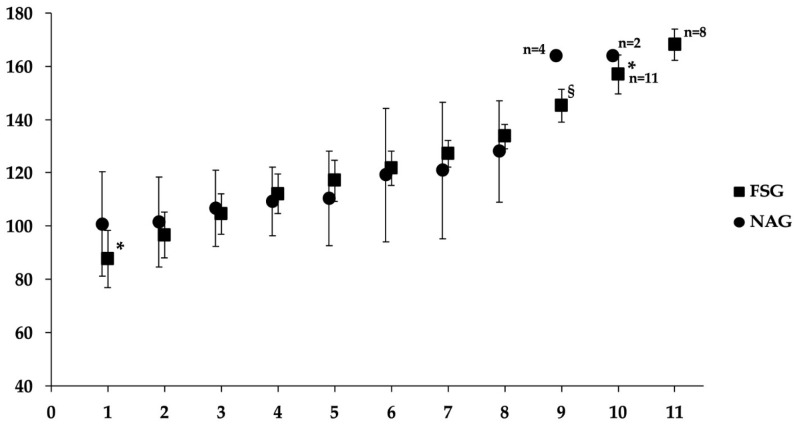
Heart Rate responses during the MSWT. The *y*-axis illustrates heart rate values achieved by the participants at the MSWT. The *x*-axis illustrates the levels of the MSWT. Bars represent the standard deviation of the mean heart rate. Significant differences between groups at specific levels are indicated by * (*p* < 0.05) and § (*p* < 0.001). FSG: Finswimmers group; MSWT: modified Shuttle Walk test; NAG: Non-athletes group.

The FSG demonstrated significantly greater exercise capacity than NAG on the MSWT, as detailed in Table 3. The FSG achieved a substantially higher total walking distance and a greater percentage of predicted capacity relative to the NAG (*p* < 0.001). Baseline hemodynamic assessments revealed that the FSG exhibited a significantly lower resting HR (*p* = 0.009), alongside significantly higher systolic (*p* < 0.001) and diastolic blood pressure (*p* = 0.019). All participants completed the 8th level (named iso-level) of the MSWT. The FSG achieved a significantly higher median walking level than the NAG, with scores of 11 (IQR: 10–11) and 8 (IQR: 8–9), respectively (*p* < 0.001). Upon peak exertion at the end of the MSWT, the FSG maintained higher HR values (*p* = 0.032), systolic blood pressure (*p* < 0.001), dyspnea (*p* = 0.015), and leg fatigue (*p* = 0.004) compared to NAG. At the iso-level (level 8), HR did not differ between groups. However, significant differences were observed in peripheral oxygen saturation (SpO_2_) and leg fatigue (Borg 0–10), (Table 3). Heart rate responses during the MSWT for both groups are presented in Figure 1. On post-exercise recovery dynamics, the FSG exhibited superior cardiovascular efficiency, characterized by a lower HR at the end of the recovery period (*p* < 0.001) and a significantly shorter total HR recovery time (*p* < 0.001).

**Figure 2:**
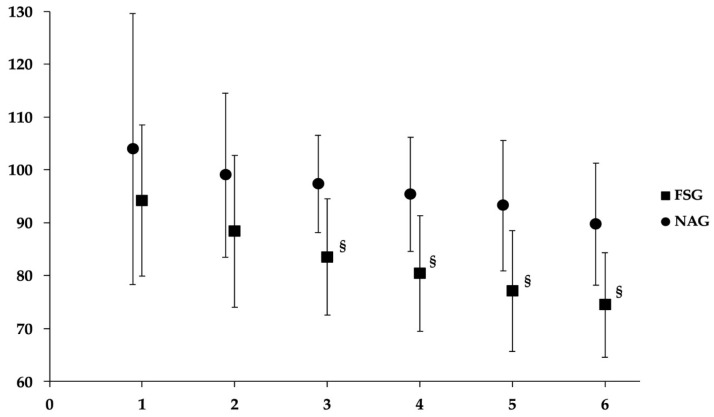
Heart Rate recovery following the MSWT. The *y*-axis illustrates heart rate values. The *x*-axis illustrates each minute (1–6) of recovery. Bars represent the standard deviation of the mean heart rate at each minute. Significant differences between groups are indicated by § (*p* < 0.001). FSG: Finswimmers group; MSWT: modified Shuttle Walk test; NAG: Non-athletes group.

Furthermore, FSG demonstrated more pronounced HR reductions throughout the entire 6-min recovery period (ΔHR_1–6_) (ΔHR: final HR–HR at each recovery minute) (*p* = 0.002 to *p* < 0.001). Heart rate recovery after the MSWT for both groups are presented in Figure 2. The cardiovascular and oxygen saturation changes (Delta: Final value–value) at each recovery minute) during the 6 min of recovery of the MSWT are presented in Table 4. Changes in heart rate were significantly different between groups for the six-minute recovery period (*p* values ranged from 0.002 to <0.001).

### Correlations Between the Modified Shuttle Walk Distance and Respiratory Function

Table 5 presents the correlations between total distance covered during the MSWT and indices of respiratory function. Total walking distance was small (r = 0.261) to large (ρ = 0.695) and correlated with the respiratory indices.

## 4. Discussion

The present study aimed to investigate respiratory function and cardiorespiratory responses in young boy finswimmers and age-matched sedentary, non-athletic peers. Our findings indicated that they have better respiratory function (Table 2) and CRF during incremental exercise (Table 3 and Table 4) compared to non-athletes.

Regarding respiratory function, the FSG exhibited significantly enhanced pulmonary parameters (FVC, FEV_1_, MVV) and superior respiratory muscle strength (MIP) compared to NAG (Table 2). These findings align with previous research by Saputri et al. [37], which demonstrated that adolescent athletes, including a subset of swimmers (n = 12), revealed higher spirometry values compared to non-athletes [37]. Furthermore, the FSG exhibited significantly higher MIP values than the NAG. These results partially corroborate the results of Santos et al. [38], who reported better respiratory muscle strength (MIP and MEP values) in swimmers (n = 25 aged 7 and 8 years) than indoor soccer players (n = 25) and sedentary boys (n = 25) (*p* < 0.05). In our study, MEP values did not differ between groups, probably attributed to the higher MEP values (104.50 ± 18.25) exhibited by the young participants in the NAG, in relation to the Santos et al. sedentary group (91.33 ± 15.77) [38]. The similarity in MEP between groups potentially stems from the healthy status of the sampled population. The ‘sedentary’ term in pediatric research rarely implies a total absence of physical exertion; rather, these populations often engage in habitual, intermittent exercise during physical education classes or at leisure time, which may account for the comparable muscle performance [6].

The discrepancy in respiratory performance between groups can support the statement that the exercise training enhances lung volumes and capacities, as previously recorded in comparative studies of athletes and sedentary peers [37,38]. Increased demands for oxygen during regular exercise training may lead to improvements in respiratory muscle performance and lung changes in expansion and elasticity [39]. The significantly higher MIP observed in the FSG suggests a robust adaptation of the inspiratory musculature to the specific demands of finswimming. Regular, high-intensity exercise stimulates improvements in pulmonary function and respiratory muscle efficiency, while the combination of aquatic resistance and controlled breathing patterns inherent in finswimming may explain the marked elevation in MIP compared to NAG [40]. The aforementioned fact is partially supported by the higher FFMI found in the FSG (Table 1), indicating higher body muscle mass.

In terms of exercise capacity, the FSG demonstrated higher exercise capacity during MSWT, achieving greater distance in meters with lower leg fatigue (Table 3). Distinct hemodynamic profiles accompanied these results; while FSG maintained a lower resting HR, they reached higher peak HR and BP values upon maximal exertion, alongside higher reported dyspnea (Table 3). Although across the second to the eighth MSWT levels, HR increased comparably in both groups (Figure 1), athletes maintained a significantly lower resting HR than non-athletes, and they reached higher peak HR values (Table 3), indicating a greater ability to reach near-maximal physiological limits. Children in both groups were within normal age-related ranges for blood pressure [41]. The elevated resting blood pressure observed within the FSG may represent a compensatory hemodynamic adaptation to chronic exercise stimuli. Such findings align with longitudinal data from youth athlete cohorts [42], suggesting that increased systemic pressure may function as a sport-specific adjustment to sustained training volumes and a concomitant rise in stroke volume.

The evidence of cardiovascular efficiency in athletes is the HR responses during and after the MSWT. This is primarily driven by coordination in the autonomic nervous system, characterized by sustained parasympathetic withdrawal coupled with sympathetic activation during exercise, followed by heightened parasympathetic activity (specifically increased vagal nerve activity) after the end of the MSWT [43]. These autonomic modulations can be observed during the recovery period, when the FSG demonstrated more pronounced HR reductions throughout the 6-min recovery period (ΔHR_1–6_) (Figure 2), a significantly shorter total HR recovery time and lower HR values at the 6th minute after MSWT (Table 3), suggesting a highly efficient transition back to parasympathetic dominance following peak effort [43]. The concomitant observation of elevated peripheral oxygen saturation (SpO_2_) and reduced leg fatigue at the iso-level of MSWT for the FSG may reflect favorable peripheral and systemic physiological adaptations [44]. Higher SpO_2_ suggests improved arterial oxygen availability, which, in the presence of diminished fatigue, indicates enhanced oxygen delivery–utilization coupling at the level of the working musculature.

We found a significant correlation (ranging from r = 0.619 to ρ = 0.695, *p* < 0.001) between the total distance covered during the MSWT and various indices of respiratory function parameters (Table 5), highlighting the importance of respiratory efficiency in the exercise capacity. These findings suggest that respiratory adaptations induced by aquatic training are fundamental determinants of exercise performance. Strengthened respiratory muscles seem to delay the onset of the respiratory muscle metaboreflex, thereby reducing the work of breathing and allowing higher workloads and percentages of predicted exercise capacity [40].

Beyond performance adaptations, FSG seems to present adaptations to the body composition as well. While basic anthropometric markers like BMI and total weight showed no significant differences, FSG exhibited a significantly higher FFMI compared to NAG (Table 1). This greater lean mass, in turn, provides the metabolic and structural foundation for the FSG to achieve greater distance during the MSWT with leg fatigue (Table 3). This significantly higher FFMI aligns with research indicating that specialized aquatic training increases muscularity, essential for overcoming resistance during high-intensity exertion [45], while lean body mass and intensity of physical activity are critical determinants of lean mass accumulation in children [46].

Finswimmers seem to reveal some distinct differences from traditional swimmers, stemming from the physiological demands of finswimming, including the use of a snorkel, which introduces additional dead space and increases CO_2_ concentration, and the frequent utilization of breath-holding techniques. These special training modalities optimize respiratory muscle strength and pulmonary volumes more effectively than in freestyle swimming [47].

Interestingly, handgrip strength did not differ between groups (Table 2), suggesting that adaptations in finswimmers are more obvious in cardiorespiratory and pulmonary parameters rather than in general musculoskeletal strength. Furthermore, in children, while handgrip strength is a practical indicator of musculoskeletal fitness and neuromuscular development, it is not associated with exercise performance [48].

### Limitations

To the best of our knowledge, this is the first study that investigated the respiratory function and cardiorespiratory responses in pediatric finswimmers. However, it is essential to acknowledge some limitations. Firstly, while the study met its power analysis requirements, the total sample size is relatively small, which may limit the generalizability of the findings to the broader population of pediatric finswimmers. Secondly, the sample consisted entirely of male children, limiting the applicability of the results to the female finswimmers. Future research should focus on comparing finswimmers with traditional swimmers, adding female athletes, to further highlight potential distinct differences, valuable when structuring special training programs that focus on enhancing performance.

## 5. Conclusions

In summary, our findings demonstrate that young boy finswimmers have better respiratory function and cardiopulmonary responses during exercise compared to sedentary, non-athlete peers. The combination of efficient cardiac autonomic control (faster HR recovery) and better respiratory capacity (higher MIP and MVV) allows young athletes to sustain higher metabolic loads with less physiological strain. These findings have limited generalizability to the broad pediatric population. However, they emphasize the importance of specialized aquatic training to address sedentary-related physiological disparities and establish a healthy cardiovascular foundation during the critical developmental years of childhood.

## Figures and Tables

**Table 1:** Anthropometric characteristics, physical activity, and handgrip strength of the participants (n = 32).

Parameter	Total Sample (n = 32)	Athletes (n = 16)	Non-Athletes (n = 16)	*p*-Value
Age (years)	9.28 ± 1.17	9.69 ± 1.44	8.88 ± 0.61	0.052
Height (cm)	141.75 ± 10.10	144.38 ± 11.62	139.13 ± 7.82	0.144
Weight (kg)	37.75 ± 11.28	40.21 ± 11.06	35.30 ± 11.29	0.223
BMI (kg/m^2^)	18.70 ± 4.03	18.98 ± 2.97	18.42 ± 4.95	0.703
FFM (kg)	27.48 ± 5.84	29.51 ± 6.52	25.58 ± 4.54	0.060
FFMI (FFM/m^2^)	13.67 ± 1.37	14.20 ± 1.25	13.13 ± 1.30	0.025
%BF	25.17 ± 8.31	25.51 ± 6.19	24.85 ± 10.01	0.826
PAQ-C (units)	2.96 ± 0.54	3.25 ± 0.26	2.68 ± 0.61	0.003
Handgrip R (kg)	12.58 ± 3.31	13.17 ± 3.43	12 ± 3.18	0.323
Handgrip L (kg)	12.91 ± 3.46	13.69 ± 4.21	12.13 ± 2.40	0.212

Values are presented as mean ± standard deviation. cm: centimeters; kg: kilograms; BMI: Body Mass Index; m: meters; FFM: Fat-Free Mass; FFMI: Fat-Free Mass Index; %BF: Body Fat Percentage; PAQ-C: Physical Activity Questionnaire for Older Children; R: right; L: left; *p*-value: differences between groups.

**Table 2:** Respiratory function of the participants (n = 32).

Parameter	Total Sample (n = 32)	Athletes (n = 16)	Non-Athletes (n = 16)	*p*-Value
FVC (L)	2.43 ± 0.63	2.67 ± 0.70	2.19 ± 0.44	0.027
FVC (%)	99.25 ± 12.37	104.25 ± 10.73	94.25 ± 12.16	0.027
FEV_1_ (L)	2.12 ± 0.51	2.34 ± 0.58	1.91 ± 0.31	0.016
FEV_1_ (%)	99.34 ± 10.39	104.56 ± 10.17	94.12 ± 7.83	0.003
PEF (L)	4.23 ± 1.14	4.64 ± 1.30	3.82 ± 0.82	0.043
PEF (%)	93.21 (88.83–97.59) *	90.50 (89.56–104.81) *	89.25 (84.90–93.59) *	0.305
MVV (L)	69.56 (59.55–79.57) *	83.62 (66.67–100.57) *	55.50 (48.76–62.23) *	0.048
MVV (%)	86.65 (78.64–94.66) *	99.43 (86.30–112.57) *	73.87 (69.62–78.13) *	<0.001
MIP (cmH_2_O)	96.37 ± 22.66	105.37 ± 24.87	87.37 ± 24.71	0.022
MIP (% pred)	95.55 ±18.15	101.29 ± 20.42	89.81 ± 13.90	0.073
MEP (cmH_2_O)	106.40 ± 21.46	108.31 ± 24.71	104.50 ± 18.25	0.624
MEP (% predicted)	97.01 ± 20.90	94.79 ± 19.49	99.23 ± 22.63	0.556

Values are presented as mean ± standard deviation or, where indicated (*), as median (interquartile range Q1–Q3). FVC: Forced Vital Capacity; L: Liters; FEV_1_: Forced Expiratory Volume in 1 s; PEF: Peak Expiratory Flow; MVV: Maximal Voluntary Ventilation; MIP: Maximal Inspiratory Pressure; MEP: Maximal Expiratory Pressure.

**Table 3:** Exercise capacity characteristics as assessed with the MSWT for the participants (n = 32).

Parameter	Total Sample (n = 32)	Athletes (n = 16)	Non-Athletes (n = 16)	*p* Value
Total walking distance (m)				
Distance (m)	666.39 ± 119.95	746.67 ± 97.22	591.13 ± 86.41	<0.001
Distance predicted (%)	67.56 ± 8.94	73.62 ± 6.65	61.85 ± 6.88	<0.001
Rest				
HR (beats/min)	79.45 ± 12	73.80 ± 9.23	84.75 ± 12.11	0.009
SpO_2_ (%)	98.81 ± 0.6	98.87 ± 0.74	98.75 ± 0.11	0.598
SBP (mmHg)	110.16 ± 13.15	119 ± 7.72	101.88 ± 11.79	<0.001
DBP (mmHg)	70.39 ± 7.56	73.60 ± 5.97	67.38 ± 7.81	0.019
End of MSWT				
HR (beats/min)	151.26 ± 23.55	160.47 ± 11.39	142.7 ± 28.6	0.032
SpO_2_ (%)	99.74 ± 0.44	99.87 ± 0.35	99.63 ± 0.50	0.130
SBP (mmHg)	125.65 ± 25.48	145.80 ± 18.67	106.75 ± 13.66	<0.001
DBP (mmHg)	76.58 ± 13.64	80.8 ± 13.30	72.63 ± 13.14	0.096
Borg Dyspnea (0–10 units)	2.48 ± 1.57	3.20 ± 0.77	1.94 ± 1.74	0.015
Borg Fatigue (0–10 units)	1.23 ± 1.68	0.53 ± 0.39	2.13 ± 1.93	0.004
Recovery (at the 6th minute after the MSWT)				
HR (beats/min)	82.35 ± 13.13	74.47 ± 9.87	89.75 ± 11.55	<0.001
SpO_2_ (%)	98.59 ± 0.62	98.47 ± 0.74	98.71 ± 0.46	0.297
SBP (mmHg)	111.48 ± 20.82	113.53 ± 24.79	109.29 ± 16.17	0.592
DBP (mmHg)	69.66 ± 9.27	69.87 ± 8.35	69.43 ± 10.47	0.902
HR recovery time (min)	5.13 ± 0.92	4.47 ± 0.68	5.75 ± 0.68	<0.001
iso-level of MSWT (Level 8)				
HR (beats/min)	130.83 ± 13.84	133.60 ± 4.56	128.00 ± 18.99	0.272
SpO_2_ (%)	99.10 ± 1.23	100 ± 0.00	98.14 ± 1.16	<0.001
Borg Dyspnea (0–10 units)	0.93 ± 1.73	1.43 ± 0.56	2.42 ± 2.04	0.081
Borg Fatigue (0–10 units)	0.93 ± 1.73	0.13 ± 0.12	2.02 ± 2.00	0.001

Values are presented as mean ± standard deviation. m: meters; HR: Heart Rate; SpO_2_: Oxygen Saturation; SBP: Systolic Blood Pressure; DBP: Diastolic Blood Pressure; MSWT: modified Shuttle Walk Test; HR recovery time: Heart Rate Recovery Time (time required for heart rate to reach baseline values).

**Table 4:** Cardiovascular and Oxygen Saturation changes (Delta, Δ) during the 6-min recovery period of the MSWT (n = 32).

Parameter	Total Sample (n = 32)	Athletes (n = 16)	Non-Athletes (n = 16)	*p* Value
ΔHR_1_	52 ± 22.52	66.26 ± 19.65	38.62 ± 16.12	<0.001
ΔSpO2_1_	1.34 ± 0.85	1.40 ± 0.98	1.28 ± 0.72	0.727
ΔHR_2_	57.38 ± 22.35	72.06 ± 19.75	43.62 ± 14.75	<0.001
ΔSpO2_2_	1.20 ± 0.81	1.14 ± 0.98	1 ± 0.55	0.194
ΔHR_3_	58.68 ± 26.26	72.12 ± 24.95	45.25 ± 20.44	0.002
ΔSpO2_3_	1.09 ± 0.85	1.31 ± 1.01	0.87 ± 0.61	0.151
ΔHR_4_	61.15 ± 26.56	75.06 ± 25.92	47.25 ± 19.34	0.002
ΔSpO2_4_	1.21 ± 0.97	1.31 ± 1.01	1.12 ± 0.95	0.595
ΔHR_5_	63.78 ± 26.80	78.18 ± 26.65	49.37 ± 18.22	0.001
ΔSpO2_5_	1.09 ± 0.85	1.31 ± 1.01	0.87 ± 0.61	0.151
ΔHR_6_	66.75 ± 27.19	80.62 ± 26.35	52.87 ± 20.56	0.002
ΔSpO2_6_	1.09 ± 0.85	1.31 ± 1.01	0.87 ± 0.61	0.151

Values are presented as mean ± standard deviation. MSWT: modified Shuttle Walk Test. ΔHR = Final HR–HR at each recovery minute; ΔSpO_2_ = Delta SpO_2_ (Final SpO_2_–SpO_2_) at each recovery minute.

**Table 5:** Correlations between MSWT distance and respiratory function (n = 32).

Parameter	Correlation Coefficients (r/ρ)	*p* Value
FVC (L)	0.627	<0.001
FEV_1_ (L)	0.627	<0.001
FEV_1_ (%)	0.475	0.007
PEF (L)	0.647	<0.001
PEF (%)	0.261 *	0.157 *
MVV (L)	0.695 *	<0.001 *
MVV (%)	0.653	<0.001
MVV (calc)	0.619	<0.001
MIP (cmH_2_O)	0.619	<0.001
MEP (cmH_2_O)	0.609	<0.001

Values are presented as Pearson’s correlation coefficients, except were indicated with (*), which represent Spearman’s rho (ρ). MSWT: modified Shuttle Walk Test; FVC: Forced Vital Capacity; FEV_1_: Forced Expiratory Volume in one second; PEF: Peak Expiratory Flow; MVV: Maximal Voluntary Ventilation; MIP: Maximal Inspiratory Pressure; MEP: Maximal Expiratory Pressure; calc: calculated.

## Data Availability

The data presented in this study are available from the corresponding author upon request. The data are not publicly available due to ethical and privacy concerns.

## Abbreviations

The following abbreviations are used in this manuscript:

20m-SRT	20-m Shuttle Run Test
% BF	Body Fat percentage
BMI	Body Mass Index
BP	Blood Pressure
CO_2_	Carbon Dioxide
CRF	Cardiorespiratory Fitness
DBP	Diastolic Blood Pressure
FEF25–75%	Forced Expiratory Flow between 25% and 75% of Forced Vital Capacity
FEV1	Forced Expiratory Volume in one second
FEV1/FVC	Tiffeneau Index
FFM	Fat-Free Mass
FFMI	Fat-Free Mass Index
FVC	Forced Vital Capacity
FSG	Finswimmers group
HR	Heart Rate
HRmax	Maximal Heart Rate
IQR	Interquartile Range Q1–Q3
MEP	Maximal Expiratory Pressure
MIP	Maximal Inspiratory Pressure
MSWT	modified Shuttle Walk Test
MVV	Maximal Voluntary Ventilation
NAG	Non-athletes group
PA	Physical Activity
PAQ-C	Physical Activity Questionnaire for Older Children
PEF	Peak Expiratory Flow
RV	Residual Volume
SBP	Systolic Blood Pressure
SD	Standard Deviation
SpO2	Oxygen Saturation
ΔHR	Delta Hear Rate
ΔSpO2	Delta SpO2
